# The Internet Is Not a Tool: Reappraising the Model for Internet-Addiction Disorder Based on the Constraints and Opportunities of the Digital Environment

**DOI:** 10.3389/fpsyg.2018.00558

**Published:** 2018-04-18

**Authors:** Alessandro Musetti, Paola Corsano

**Affiliations:** Department of Humanities, Social Sciences and Cultural Industries, Università Degli Studi di Parma, Parma, Italy

**Keywords:** digital environment, internet addiction, internet use, cognitive ecology, virtual reality

## Historical overview

The Internet was born in the United States in the second half of the twentieth century; it was initially used for military purposes but has since become a powerful instrument for nonmilitary use, including the exchange of information all over the world, thanks to the introduction of tools such as the web browser. *From the start*, the World Wide Web assumed several functions (e.g., recreation, education, and business) but preserved a private dimension. To connect, people needed access to an Internet-connected computer, which represented a separation from real life, or a virtual reality. A video-terminal device helped these people to immerse themselves in salient but virtual images and sounds; this immersion could induce symptoms such as dissociation (Schimmenti and Caretti, 2010). In the 1990s, scientists developed a conceptualization of the misuse of the Internet and of Internet-addiction disorder (IAD) that was coherent with their conception of the Internet as virtual reality. The strongest criterion for distinguishing healthy Internet use from misuse was connection time; this criterion was supported by several empirical studies regarding its relationship with psychopathological symptoms (Young, 1998; Quayle and Taylor, 2003; Musetti et al., 2016, 2017).

However, over the last two decades, Internet use has given rise to global sociocultural changes and has had important implications for the functioning of people's minds (Clowes, 2015). Today, digital and connectable tools such as smartphones are powerful, very small, portable, and (thanks to WiFi and cloud technology) able to store a great deal of salient information about people's lives. These tools thus assume the function of an e-memory (electronic memory) by expanding cognitive memory (Clowes, 2015). Virtual reality is no longer synonymous with the Internet, so there is a need to reformulate the conceptualization of the Internet by taking into account its evolution. The extent of digital information in every sphere of people's lives has caused the integration of the Internet into the cognitive tasks people perform in their daily routines, leading to the consideration of the Internet as part of an extended concept of cognition (Smart et al., 2017). The concept of the Internet as a tool to connect to a virtual reality that is separate from the real world is no longer current, so a new concept of the Internet that takes its environmental features into account is needed. This concept is in line with Floridi's (2014) idea of an infosphere that shapes people's reality. The conceptualization of the Internet as an environment rather than as a tool leads to the reformulation of IAD theory. If the Internet is not just a tool to be utilized, the theoretical model of IAD cannot be based on behavior connected to its overuse, misuse, or abuse.

Based on this opinion, we present arguments in favor of reconsidering the Internet as an environment rather than as a tool. In the following section, we explore the Internet's role in cognitive ecology, as well as the inadequacy of treating the Internet as a tool and thus of the current Internet-addiction model.

## The internet and cognitive ecology

One conceptualization that could help explain the idea that the Internet is a superstructure within which people operate is that of cognitive ecology (Smart, 2017), which has been defined as “the multidimensional contexts in which we remember, feel, think, sense, communicate, imagine, and act, often collaboratively, on the fly, and in rich ongoing interaction with our environments” (Tribble and Sutton, 2011, p. 94). Today's society is digital (Lupton, 2015), and the Internet represents the main part of its cognitive ecology. In the theory of situated cognition (Robbins and Aydede, 2009), cognition is embodied (Gallagher, 2005), embedded (Rupert, 2004), extended, and distributed or collective (Smart et al., 2017). These theories reconceptualize cognition; instead of the classical, individualistic and intra-brain conception of cognition, these theories take into account the relationships among the brain, the body, and the environment to determine the functional products of the mind (Smart et al., 2017). Thanks to the Internet's development (in terms of devices, apps, and social platforms), it can be seen as the principal structure of embodied, embedded, extended, and distributed cognition. Proponents of the embodied-cognition thesis claim that extra-neural bodily factors shape the course of cognitive processing (Anderson, 2003; Shapiro, 2007, 2011). Mobile or wearable devices such as smartphones are today part of people's daily engagements, and they allow continuous online access, which shapes the course of their daily activities and interactions (Smart et al., 2017). By contrast, proponents of the embedded-cognition thesis claim that the extra-organismic environment plays a role (although not a constitutive one) in cognitive states and processes (Rupert, 2004), thus reallocating cognition to within biological boundaries (Smart et al., 2017). The Internet can be inserted within this vision of cognition. For example, augmented reality devices (Smart et al., 2017) such as Google Glass can enrich the sensory experience and have repercussions on cognitive processes. Advocates for the extended-cognition thesis claim that cognitive processes supervene on the relation between a cognitive agent and the social environment in which that agent is situated (Smart et al., 2017). Internal (biological) structures and external devices work in a pair relationship in which biological structures can perform the same operations as external factors (see Clark and Chalmers, 1998) or in a complementary relationship in which external devices can perform operations that biological structures cannot, and vice-versa (see Sutton, 2010; Heersmink, 2015, 2016). The debate regarding the parity or complementarity of the Internet and the brain has not yet been resolved (Smart et al., 2017), and it is not our aim to discuss that issue here. What is important in this context is that Internet devices are so widespread in the social environment that they are the principal external factor through which people's brains relate to and structure external representations; these devices have thus become integrated in people's cognitive architectures (Halpin et al., 2010). Consider the examples of how the use of GPS has modified people's spatial navigation, including its important impact on the neural mechanisms of spatial cognition (Maguire et al., 2000), or considering how Facebook use shapes the representation of the self, including an important impact on the self-concept. This effect is not merely about the interaction between a cognitive agent and environmental devices or about the scaffolding function that external factors have within the mind. The Internet is more than just a scaffold that guides and integrates the mind as it performs functions that the mind cannot accomplish alone (Sterelny, 2010). Rather, people created the Internet to meet people's needs, and the Internet's functions, such as that of e-memory, have changed the ways in which people remember and behave in the world (i.e., a person can recover remote information without having to store every piece of information from day to day). The Internet has changed people's brain structures, which have in turn evolved in such a way as to change how the Internet meets new needs (Clowes, 2013). This view requires consideration of the Internet as an extended function of the mind, including its actual effects on the development of the brain's circuits. In a similar vein, the advent of cooked food changed not only people's tastes but also their digestive functions and the structures of their jaws and teeth; it thus had repercussions on environmental adaptation and species conservation (Wrangham, 2009; Sterelny, 2010). The last thesis regarding the Internet's crucial role is that of distributed cognition. This thesis relates to the cognitive processes (e.g., focusing, reasoning, remembering, and problem-solving) that a collection of individuals share. Again, the Internet has allowed people to take advantage of a huge network of geographically distributed individuals who process cognitive operations at the same time and on the same issue. This opportunity boosts collaboration, information exchange, and the coordination of collective efforts and collective decision-making (Chi et al., 2008; Chi, 2009; Smart et al., 2017). These theories of cognition are today a matter of debate. Some authors have preferred one vision over others; others have considered the theories to not be mutually exclusive and to instead by various integrated aspects of cognition. In *the article*, we want to underline that, irrespective of the vision that one embraces, the Internet represents a fundamental part of cognitive processing. It not only boosts cerebral operations but also shapes, modulates, and changes neurobiological structures, functioning, and development; the Internet is also, in turn, shaped and developed in a process that resembles a spiral of mutual influence toward ever-higher steps of development.

In this sense, a view of the Internet as a mere tool to be utilized functionally or dysfunctionally, as in the model of Internet addiction, is reductive in this era. Thus, considering the Internet as a digital environment that encloses and characterizes cognitive processes is more useful for understanding the phenomenon that we are studying.

## The internet as more than a tool

Consider the people of the nineteenth century, who began to deal with great technological changes (due to the Second Industrial Revolution). The invention of the train, for example, represented a substantial change in the connection between long distances and/or in the amount of people or material carried. People also had to learn to use trains by acquiring new behaviors such as buying tickets and waiting for the departure time; these behaviors could be functional or dysfunctional (examples of the latter include buying an expensive ticket or getting on the wrong train). Although the train was intended as an instrument for traveling to a destination, its growth into a global network and its various functions (industrial, civil, and military) fostered the sociocultural revolution of the 1800s. The train changed the way people thought about industry; thus, in the nineteenth century, the bourgeoisie affirmed its power, and science and literature became more liberal. In other words, what began as a mere instrument evolved into an environmental change that people had to adapt to.

The example of the train concretely describes the difference between a tool and a sociocultural environment. The dynamics of the person–tool interaction have been thoroughly studied and represent the basis for the strong Vygotskian psychological tradition (Luria and Vygotsky, 1992). According to this tradition, children organize their behavior by learning to use tools or through external stimuli (Vygotsky, 1997). For example, a child might pay attention to a tool and then name the tool; the name of the tool thus becomes a word in the child's internal speech, thus inducing a new step in the child's reasoning and language functions (Bodrova et al., 2011). This explains how the development of higher brain functions is mediated by the utilization of tools, a view that fits well with the thesis of embodied cognition, according to which external tools shape the course of cognitive processing. It also fits with the thesis of scaffolding cognition, according to which external tools drive cognitive functioning. Within the latter conceptualization, the Internet can be seen as a tool through which people interact and whose use shapes the course of their cognitive processing. However, this view is reductive because it does not take into account the extra-brain operations that the Internet can provide but that the brain cannot. For instance, in the scaffolding view, people can interact with a social platform that reminds them of a salient episode that occurred in their past, thus shaping their emotional reactions and/or thoughts. However, in this view, social-platform interaction does not allow for the improvement of memory systems to provide a better ability to remember salient episodes from the past. Rather, the social platform is seen as a context inside which a limited memory system can take advantage of externally stored information, thus optimizing its work and allowing cognitive resources to be delivered to other processes. In other words, although the Internet—at least in its embryonic form, when recreation was the main online activity—was once considered a tool that shaped and mediated cognition and behavior, today, it is considered an environment that characterizes the people of today. To return to the example of the train, at the beginning, it was considered to be a tool for enhancing travel, but after a few decades, it began to shape the environment that characterized people in the industrial era. Interestingly, Floridi (2014) explained how tools, in addition to being utilized to boost behaviors, have also changed the sociocultural fabrics of various eras, thereby marking the evolution of humanity. The use of bronze (starting in 3000 BC) changed the prehistoric world into the Bronze Age. Similarly, today, people are part of an information society (also known as the infosphere) and can access whatever information they lack (e.g., facts about laws, politics, or science), meaning that there are no boundaries between their online and offline lives—a state known as “onlife” (Floridi, 2014).

As the reader may have noted, the arguments in favor of considering the Internet as an environment have multiplied and advanced. It is important to underline this vision here because the classical model and the resulting research into IAD are based on an obsolete conceptualization of the Internet as a tool.

## Reappraising internet-addiction disorder

Over the last three decades, the literature on this phenomenon has been abundant, but scholars have not reached an agreement on which criteria must be focused on when determining the dividing line between pathological or nonpathological Internet use (Musetti et al., 2016). The main models of Internet-related pathologies retrace those of other addictions (Young, 1998). If the theorists of IAD do not consider the Internet to constitute the current information society, they risk pathologizing a normal behavior, similarly to what happened for new addictions (as with new terms such as “shopaholic” or “workaholic”; see, e.g., Billieux et al., 2015). Without the environmental framework of the Internet, the theorization of pathological Internet use is limited to a reductive list of potentially problematic behaviors (Schimmenti, 2017), such as using the Internet for pornography or gambling. It is noteworthy that the *DSM-5* does not resolve this impasse, as it does not mention IAD; the only related disorder, online gaming disorder, is inserted in a section regarding diagnoses that require further study (American Psychiatric Association, 2013). The seven symptoms of IAD in the classical model are withdrawal; tolerance; concern over Internet use; heavier or more frequent Internet use than intended; centralized activities to obtain more from the Internet; loss of interest in other social, occupational, and recreational activities; and disregard for the physical or psychological consequences of Internet use (Young, 1998). These criteria must be present for at least 1 year. Clearly, these criteria are not applicable to the vision of the Internet as an environment. If the Internet constitutes the social fabric, it becomes impossible to withdraw from it, making it impossible to be concerned over Internet use; it likewise becomes impossible to focus on obtaining the Internet. In particular, the criterion of “heavier or more frequent use of the Internet than intended” lacks a comparative parameter in the environmental view of the Internet. How much Internet use is normal if the Internet is ingrained in every part of people's lives and also extends their cognition? In the environmental view, considering the amount of time spent online to be a pathological criterion would mean seeing the entire information society as pathological. Moreover, and paradoxically, a rehabilitation treatment based on this criterion would be centered on reduced Internet access, thus limiting the use of extended and collective cognition (Smart et al., 2017), which could have important repercussions with regard to social adaptation that, in turn, would favor an increase in other pathological criteria, such as withdrawal from social occupation or recreation.

## The internet as a social environment

Our position is that the classical IAD model should be reformulated to match the vision of the Internet as a social environment. First, researchers must determine whether it is actually possible to be addicted to the Internet. In other words, can people become addicted to their social fabrics? Perhaps it is possible for a person to manifest difficulties or abnormalities when adapting to a social environment. In a similar vein, new models should ignore utilization-related criteria and instead focus on the symptoms that indicate social maladaptation, which may resemble manifestations of known symptoms such as dissociation, depression, anxiety, and personality disorder (Musetti et al., 2018). If this new focus were applied, a question would need be raised about what preexisting pathological conditions would predispose a person to have difficulty adapting to an environment (Caplan, 2002). Considering the Internet as the current socio-cognitive environment, a person's preexisting intra-brain features could favor the success or failure of the adaptation process. In an interesting model, scholars have suggested that maladaptive cognitions precede the symptomatology of IAD (Davis, 2001; Taymur et al., 2016), thus underlining the comorbidity of IAD with heterogeneous psychopathological diagnoses (Orsal et al., 2013). A child presenting with an attention disorder will have some difficulty adapting to a school environment and to a social network of peers, and this difficulty will often impair the development of the child's intellectual and other cognitive functions. Similarly, a person who is cognitively poorly equipped could fail to take advantage of the Internet's contextual affordances (Ryding and Kaye, 2017). This could result in the unsuccessful extension and/or distribution of cognition processes, with repercussions for the person's cognitive development and risks of pathological adaptation to the digitized environment. A similar view could be used in studies on the appropriate treatments for cognitively predisposing features and to help explain the adaptation processes.

## Conclusion

We are in favor of treating the Internet as a social environment in which a cognitive agent exists. Our proposal is that Internet use should not be seen as a mere instrumental action to achieve a goal (and which could be functional or dysfunctional); rather, we propose treating Internet use as an action situated in the digital context, as part of a system with a proper structure and rules. Considering the concept of the Internet as a social environment, the classical IAD model should be reformulated, as its implications are obsolete and misleading when applied to studies on the pathological population or on potential treatments.

## Author contributions

AM: devised and structured the paper; PC: contribute to development and deep revision of the work, with literature analysis and agreement for final approval of the paper.

### Conflict of interest statement

The authors declare that the research was conducted in the absence of any commercial or financial relationships that could be construed as a potential conflict of interest.
